# Ambulance Work, the Public, and the National Guard

**Published:** 1915-01-02

**Authors:** 


### AMBULANCE WORK, THE PUBLIC, AND THE NATIONAL GUARD.

One of the consequences of the war has been a greatly increased interest in first-aid and ambulance work. In the early days, classes crowded with ladies sprang up on every side; medical instructors explained to anxious audiences the mysteries of the triangular bandage and the methods of making a poultice; and the sale of books carrying the stamp of the red cross was enormous. More recently the click of the knitting needle has replaced the differential diagnosis between fracture and dislocation. When we say that, while both are good, we should, had we to make a choice, elect for the knitting, we have no desire to disparage the earlier activity. On the contrary, we think its influence is likely to prove a very beneficial one. Certainly we hope that the first-aid ladies will not have any wide opportunity of practising their newly acquired skill on sick and wounded soldiers, for this is only another way of saying that we trust this country is not to become the theatre of active warfare. All the same, classes in first-aid have their value, and we take it that this value is an indirect rather than a direct one. Occasionally, perhaps, and where the instruction is followed by systematic training and practice—in the police, for example—it produces highly valuable capacities. But whatever other end first-aid classes promote they certainly help to spread the conviction that to give help efficiently needs more than kindness of heart and good intentions.

The basis of efficiency, here as elsewhere, is knowledge and training, and this truth is made manifest to all who attend a course of instruction in first-aid. A second result of such a course must be a realisation of the enormous importance of wise action at the outset—as, for example, immediately after an injury. There may be added to these an appreciation of the difference in comfort, as well as in safety, which skilful handling, as compared with unskilful, makes to a sick or injured man. If these lessons are widely learned they must inevitably increase in the community a recognition of the claim that efficient first-aid and ambulance work are demands which every humane person would wish to satisfy. That every individual member of the public, or indeed any large proportion of the public, can be efficient for the task is simply impossible. But an educated public opinion—and first-aid classes are helping to form this—may be led to see that it is the duty of the municipal authorities everywhere to provide both men and apparatus fit to meet the duty recognised by the community.

### Doctors and the National Guard.

We venture to say that their indirect influence can be detected both in the more sensitive attitude of our municipal authorities and in the splendid response recently given to the request for motor ambulances for the conveyance of wounded soldiers to the hospitals. To have helped in these directions is something of which the first-aid classes have the right to be proud.

There is just now another opportunity which, if rightly used, may extend both the knowledge and the appreciation of efficient first-aid and ambulance work. We refer to the formation of the National Guard. It is obvious from what has been occurring in London during the last few weeks that the National Guard is a serious movement, and that it is likely to attract large numbers of men. Clearly there must be associated with each corps an adequate supply of medical officers, and we take it that equally there must be arranged some form of ambulance equipment, including the necessary "bearers." In other words, bodies of men, in London and elsewhere, will be brought into a position where they will realise, and some of them for the first time, the importance of first-aid and ambulance assistance. Further, some of the members will be drawn from classes largely protected from risks of accident and injury, and, at the same time, of considerable social importance. The workman, either in his own person or in those of his fellows, is familiar with accidents and resulting journeys to hospital. But the merchant, the lawyer, the banker, mainly know such experiences only by hearsay. In such ranks ambulance instruction has therefore a special opportunity. We trust this opportunity will be wisely utilised.

If, as may be anticipated, the National Guard is to have a medical service, there is particular need that it be an experienced one. The members of the Guard will be men of middle age and upwards. Many of them have been accustomed to a life in which active muscular exertion has played but an insignificant part. They will now be subjected to the strain involved in drilling, marching, rifle-firing, and so on. Such a combination of events is highly likely to discover weak spots, the existence of which had previously been unsuspected. It is not therefore the mere accidents incidental to outdoor activities which the medical service of such a corps ought to be competent to meet; in addition, there must be expected the less obvious evidences which announce strain in excess of the vital resources of middle-aged men. The recognition of these and their true appraisement require experienced judgment. Often, too, for such ends it is advisable or even necessary that the medical officer should see any symptoms of disorder for himself, and should not be entirely dependent on descriptions afforded by the patient or other unskilled observers. These latter may easily, though unconsciously, either exaggerate or minimise the facts, and thus their stories may be misleading. The medical consideration in reference to such a body of men is not merely that they shall be "fit" when the training commences, but that they shall remain "fit" when subjected to unaccustomed activities. Signs of impending failure which might easily escape an inexperienced eye would be confidently recognised and interpreted by a riper judgment. Therefore the new corps should have the directing influence of competent medical authority.

